# Investigation of Differences in Tobacco Use Language Between Groups: Corpus-Assisted Analysis

**DOI:** 10.2196/86593

**Published:** 2026-07-15

**Authors:** Iona Fitzpatrick, Xinmei Sun

**Affiliations:** 1 Tobacco Control Research Group, Department of Health, University of Bath, Claverton Down, Bath, BA2 7AY, United Kingdom; 2 Lingustics and the English Language, School of Social Sciences, Lancaster University, Lancaster, United Kingdom

**Keywords:** corpus linguistics, health information, tobacco use, commercial determinants of health, health information

## Abstract

**Background:**

The variation of language concerning tobacco products and tobacco use is known to impact the understanding of related risks and influence behaviors including use uptake and product cessation. Transnational tobacco companies can use such complexities to change the acceptability of tobacco use and influence public understanding of related risks. These changes, in turn, impact tobacco use behaviors. Looking at variations in the language used by different groups can therefore offer helpful insights into tobacco use cultures and make imbalances of information between groups plain. This paper examines the language of tobacco use, specifically smoking, across a sample of health organizations (the National Health Service, the World Health Organization, the National Institute for Health and Care Excellence, and the Centers for Disease Control and Prevention), the tobacco industry (British American Tobacco and Philip Morris International), and in “general English.”

**Objective:**

Through corpus-assisted analysis, the study aims to illustrate differences in tobacco use and tobacco user characterizations by 2 transnational tobacco companies, health organizations, and users of general English. The study assesses the possible implications of these differences for public health.

**Methods:**

We queried 4 bodies of text (corpora) from 3 different groups; 2 of these corpora were preexisting corpora of general spoken and written English in the United Kingdom and the United States. The remaining 2 sampled tobacco-related documents from 2 transnational tobacco companies and from the United Kingdom and international organizations with a focus on health between 2003 and 2023. The 2 sampled corpora contained 1355 documents and 10,023,538 words. We used the corpus analysis software LancsBox (Lancaster University) to identify variations in characterizations of tobacco users and tobacco use behaviors between these groups, using the stems “smoker*” and “smok*.”

**Results:**

Frequency and collocation analysis showed clear differences in how the 3 groups described smokers and smoking, with only limited overlap in the terms they used. Only 7 of 23 unique categorizations of “smoker*” were shared. There was a significant association (*P*<.001) between individual corpora and singular or plural noun forms. Health organization texts more often used clinical and population-focused classifications, whereas tobacco industry texts more often framed smokers as consumers or people involved in legal disputes. General English corpora showed the widest range of labels for tobacco users.

**Conclusions:**

While there was some overlap in terminology used between corpora, the most common categorizations in each corpus were highly varied, showing very little shared language between groups in their descriptions of either tobacco users or use behaviors. This variance indicates that these groups may not share the same sense-making resources related to tobacco use, which may render information flows around tobacco vulnerable to distortion.

## Introduction

Tobacco is a leading cause of preventable death globally, killing more than 8 million people annually [1]. Despite sustained international efforts to curtail the manufacture, sale, and use of tobacco, tobacco use is not in universal decline [2]. The rapidly evolving landscape of tobacco and nicotine products, including e-cigarettes, heated tobacco products, and nicotine pouches, presents an ongoing challenge to policymakers, regulators [3], and consumers [4] alike, who are faced with an ever-expanding range of nicotine and tobacco products and product-based information.

The volume of available products and the proliferation of brands driven by the tobacco industry are known to confuse and mislead consumers [3,5] and contribute to the spread of misinformation regarding usage risks [6]. At the same time, language relating to tobacco varies significantly across user categories—for example, between “non-smokers” and “never-smokers” in their personal narratives, including descriptions of risk and addiction associated with product use [7]. Some features of newer nicotine and tobacco products, such as flavorings, connect nicotine and tobacco use to the relatively innocuous consumption of sweets [8]. It is through practices like this that what it means to use tobacco and to be a “smoker” is increasingly contested [9]. Different groups also vary in their understandings of risk and in their ability to overcome structural constraints in their tobacco use behaviors [5]. Individuals living with mental ill health, for example, are especially vulnerable to misconceptions of harm relating to nicotine and tobacco product use [10].

Uncertainty surrounding nicotine and tobacco products, as well as the risks associated with their use, is not exclusively a problem for consumers. Efforts have been made to shift language away from terms that are imprecise, inherently negative, or derisive [11,12]. In public health and scientific communication, for example, the rise in calls for “person-first” language is notable [13,14], but the route to consensus is not clear. Some alternatively advocate for a focus on personal agency in reporting on tobacco use [15]. The shift to person-first language has not been universally adopted and has come under criticism for being overly simplistic and for perpetuating stigma [16].

Health-related language used by the scientific community and the public does not consistently overlap, sometimes relying on outdated conceptualizations of ill-health [17] and tobacco use [18]. Policymakers and regulators share in these dilemmas. Lempert et al [19] highlighted disparate, overly specific definitions as a problem for effective regulation as early as 2014. It is not surprising, then, that existing research has highlighted a “jingle-jangle” problem [11] and describes the muddled terminological landscape as a hurdle in “attempting to integrate evidence internationally” [20].

Guidance published in 2024 by Maddox et al [21] suggests that better alignment of language on the part of public health could help counter the narratives of powerful industries more effectively, including by calling out “co-opted and misleading terms and phrases” and through the use of “person-centered language.” Both of these strategies, they argue, could serve to help in “disarming” harmful industries like the tobacco industry.

As tobacco use is both an individual-level behavior and a socially shaped practice [22], intertwined with cultural values and expressions of identity, we make use of corpus-assisted methods to provide a quantitative evidence base for the examination of tobacco use discourse [23]. Previous applications of these methods have highlighted the importance of linguistic choices in the negotiation of responsibility [24] and the cultivation of stigma [25] in the context of health communication.

Our querying of 2 tailor-made tobacco-focused corpora and 2 ready-made corpora of general English explores and clarifies the linguistic variations surrounding tobacco use status and tobacco use behaviors among groups (the tobacco industry, health organizations, and users of general English).

## Methods

### Overview

We applied methods from corpus linguistics to investigate language variance between three preselected groups with converging interests in tobacco control. The 2 largest transnational tobacco companies, British American Tobacco (BAT) and Philip Morris International (PMI) [26], were selected to sample tobacco industry language. We selected health organizations from the United Kingdom, the United States, and internationally to sample institutional health communication: the National Health Service (NHS), the National Institute for Health and Care Excellence (NICE), the Centers for Disease Control and Prevention (CDC), and the World Health Organization (WHO). These English-language samples matched the English-language balanced corpora we chose: the 27 British National Corpus 2014 (BNC2014) [27,28] and the Corpus of Contemporary American English (COCA) [29]. We used both the written and spoken components of the 2 corpora, spanning genres such as informal speech (transcribed to text), newspapers, fiction, television scripts, and social media. To compile new bodies of language for querying, we constructed detailed search queries for the tobacco industry and health organization corpora.

### Setting the Frame and Searching for Sources

The time frame for data collection was set between January 1, 2003, and December 31, 2023. The period from 2003 to 2023 was marked by huge upheaval in both the tobacco consumer product landscape and information ecosystems. In May 2003, member states of the WHO adopted the WHO Framework Convention on Tobacco Control. Although it did not enter into force until 2005, the adoption marked a concerted effort “for the promotion of public health” on a global scale. Simultaneously, a consistent consolidation of tobacco companies meant that the tobacco industry had also undergone some major shifts—from a number of small and medium enterprises to just a few global powerhouses [18], of which PMI and BAT are the largest.

The 2 ready-made, balanced corpora of English—BNC2014 and COCA—were chosen for the insights they might offer into the way a “general” English-speaking public talks about tobacco products and tobacco use behaviors. The BNC2014 is a 100-million-word collection representing British English, with texts dating between 2007 and 2020, and the COCA is a 1-billion-word collection representing American English, with texts dating between 1990 and 2019.

For feasibility purposes, the construction of the Tobacco Industry Corpus (TIC) was restricted to BAT and PMI, the 2 largest transnational companies based on 2021 market value [26]. The search parameters for the TIC included web pages and documents published by BAT and PMI during the set time frame. The search query was designed to capture content containing multiple terms related to smoking status and smoking behavior. Asterisks indicate “wildcard” searches, which capture various word endings following a given stem:

smok* OR quit* OR cessation OR switch* OR vap* OR experiment* OR relaps* OR “us* NRT*” OR “dual” OR addict*

Table 1 provides examples of terms captured by different parts of the search query. We conducted three separate searches: (1) a Google site search, (2) using the search function within the two tobacco company websites, and (3) searching web archive tools—the Internet Archive [30] and Archive Today [31]—to collect backdated content not captured in searches one or two. All documents were available in the public domain.

**Table 1.** Example terms captured by wildcard search queries^a^.

Search term	Examples of terms captured
smok*	smoker, smokers, smoke, smoking, smoked
quit*	quitter, quit, quits, quitting
switch*	switcher, switch, switching, switched
vap*	vaper, vape, vaping, vaped
experiment*	experimenter, experiment, experimentation
relaps*	relapse, relapsed, relapsing
us* NRT*^b^	use NRT, using NRT, using NRTs
dual	dual users, dual use, dual usage, dual e-vapor combustible users
addict*	addict, addicted, addiction

a Asterisks indicate “wildcard” searches, which capture various word endings following a given stem.

b NRT: nicotine replacement therapy.

The search parameters for the Health Organizations Corpus (HOC) were webpages and documents published by 4 major health organizations: NICE, NHS, CDC, and WHO. For the HOC, we added the following to the previous search query to refine the content returned to be about tobacco:

AND (tobacco OR smok* OR vap*)

The study conducted two searches: (1) a Google site search of the named organizations and (2) the WHO Institutional Repository for Information Sharing [32]. The study excluded workbooks, research papers, replacement guidelines, and any draft documents.

### Preprocessing and Compiling Corpora

When compiling both corpora, lists of returned results were saved as structured CSV files containing metadata, which included URLs, webpage headings, and publication dates. This list was manually filtered to exclude job advertisements, pages spotlighting named individuals, and pages or documents in which search terms only appeared in legal disclaimers. Region-specific or geographically local content pages were also excluded, as our focus was on organizational rather than local characterizations. Documents or webpages consisting mainly of figures or videos were also excluded from both the TIC and the HOC for reasons of feasibility. Descriptions of smoking status and smoking behavior were our primary focus, so we manually filtered the data files to ensure documents focusing on individuals (profiles or personal stories) were not included in the corpora. The TIC includes multiple document types directed at different audiences, including investors and consumers. This was intentional, as the aim was to capture the tobacco industry’s discourse at the organizational level rather than to compare communication strategies across internal genres.

After manual filtering and the application of the exclusions listed above, we used Python (Python Software Foundation) scripts to process the CSV files. The scripts included routines for downloading linked PDF documents, extracting and cleaning text from PDF documents using the *pdfminer* library [33], and exporting web-based content as individual plain text (.txt) files. Annotations of page numbers were included in the files to preserve cross-references to the original PDF documents. The author and publication year were included in the given file names. The sources used to compile the corpora are available via the University of Bath Research Data Archive [34]. Once processed, corpus documents were imported into LancsBox using automatic part-of-speech tagging, with LancsBox (Lancaster University) default settings applied.

Table 2 shows the composition of the 2 tailor-made corpora, the TIC and the HOC.

**Table 2.** Composition of tailor-made corpora.

Organization and document type	Files	Unique words (types)	Total words (tokens)
TIC^a^			
BAT^b^			
Annual and sustainability reports	40	32,599	2,893,751
Webpages	170	8306	127,129
Other documents	35	19,078	1,123,665
Total	245	59,983	4,144,545
PMI^d^			
Annual and sustainability reports	24	20,393	1,415,877
Webpages	406	15,031	457,267
Other documents	102	19,569	629,801
Total	532	54,993	2,502,945
Corpus total	777	114,976	6,647,490
HOC^e^			
NICE^f^			
Documents	15	5966	94,513
Webpages	20	2254	21,422
Total	35	6641	115,935
NHS^g^			
Documents	1	2173	11,186
Webpages	23	1861	13,790
Total	24	3189	24,976
World Health Organization			
Documents	124	69,175	2,428,207
Webpages	120	7239	81,256
Total	244	69,947	2,509,463
CDC^h^			
Documents	69	33,757	583,435
Webpages	206	9406	124,628
Total	275	37,012	725,674
Corpus total	578	116,789	3,376,048

a TIC: Tobacco Industry Corpus.

b BAT: British American Tobacco.

c PMI: Philip Morris International.

d HOC: Health Organizations Corpus.

e NICE: National Institute for Health and Care Excellence.

f NHS: National Health Service.

g CDC: Centers for Disease Control and Prevention.

### Analytical Approach

The data were analyzed using a corpus-assisted discourse approach, which combines quantitative corpus techniques with qualitative discourse interpretation to examine patterns in large collections of text [35]. In practical terms, statistical measures were used to identify potentially meaningful patterns in how people talked about tobacco use status and tobacco use behavior, and then those patterns were inspected in their immediate context to interpret what they were doing in the discourse.

The analysis drew on 2 complementary techniques—collocation analysis and concordance analysis. For collocation analysis, we examined which words occurred unusually often near a target term to identify recurring associations in the corpus. Collocational strength was measured through mutual information using LancsBox (TIC, HOC, and BNC2014) and English-corpora.org (COCA). Collocations were calculated within a window of 5 words to the left and 5 words to the right of the target term; this is the norm in corpus research and has been shown to be productive in corpus-based discourse studies [36]. A minimum raw co-occurrence frequency of 10 was used to keep the number of collocates manageable for detailed qualitative analysis. Common function words (eg, the, and, of) were excluded using the Natural Language Toolkit’s stop-word list [37]. We then used concordancing to interpret these quantitative patterns by viewing every occurrence of a search term in its immediate context, supporting qualitative examination and interpretation of differences and similarities in discourse.

### Ethical Considerations

Patient engagement or involvement was not applicable or relevant to the project, as it made use of documents available in the public domain. Institutional ethical clearance was provided by the University of Bath Biomedical Ethics Committee with approval EP18/19 004. The research was conducted in accordance with the guidelines set forth in the Declaration of Helsinki.

## Results

### Frequencies

The singular form “smoker” and the plural form “smokers” were analyzed separately to assess whether any of the corpora showed strong habits of reference. Table 3 presents the raw and relative frequencies of the terms “smoker*” as a noun and “smok*” as a verb in each corpus.

**Table 3.** Frequency of “smoker* N*” and “smok* V*” in each corpus^a^.

	Raw and relative (per million) frequency			
	TIC^b^	HOC^c^	BNC2014^d^	COCA^e^
smoker* N*				
smokers N*	4924 (7.39)	7577 (22.44)	458 (4.48)	4120 (4.15)
smoker N*	936 (1.40)	648 (1.92)	206 (2.01)	2355 (2.37)
smok* V*				
smoke V*	2042 (3.06)	2749 (8.14)	1138 (11.12)	43,675 (43.98)
smoking V*	1126 (1.69)	1696 (5.02)	1135 (11.09)	30,393 (30.61)
smoked V*	272 (0.41)	1330 (3.94)	475 (4.64)	8866 (8.93)
smokes V*	17 (0.03)	130 (0.39)	131 (1.28)	2278 (2.29)

a Asterisks indicate “wildcard” searches, which capture various word endings following a given stem, while the asterisks after V and N indicate "smart searches" where the term is tagged as either a Noun (N*) or VERB (V*).

b TIC: Tobacco Industry Corpus.

c HOC: Health Organizations Corpus.

d BNC2014: British National Corpus 2014.

e COCA: Corpus of Contemporary American English.

A chi-square test showed a significant association between corpus and noun form (singular vs plural; n=21,224; *χ*²_3_=1982.01; *P*<.001; Cramér’s *V*=0.31), indicating a large effect. Bonferroni-adjusted pairwise comparisons (*α*=.0083) showed significant differences between all corpus pairs (all *P*≤.007). HOC had the highest proportion of plural forms, with the largest pairwise difference observed between HOC and COCA (φ=0.35). By contrast, the difference between BNC2014 and COCA, although statistically significant, was negligible in magnitude (φ=0.03). The predominance of plural forms in the HOC constructs smoking as a population-level phenomenon, while comparatively greater use of singular forms elsewhere individualizes the smoker.

The distribution of verb forms also differed significantly across corpora (n=97,453; *χ*²_9_=1173.18; *P*<.001); however, the effect size was small (Cramér’s *V*=0.06). TIC showed the highest proportion of the base form “smoke,” whereas HOC favored the past tense “smoked,” consistent with the retrospective orientation of clinical and epidemiological reporting.

### Collocation

To explore how smoking status and behavior were constructed across corpora, we investigated the semantic themes of collocates of “smoker*” as a noun and “smok*” as a verb using the complex queries “smoker* N*” and “smok* V*.” Themes were generated through an iterative examination of concordance lines of the identified collocates, with provisional categories developed, reviewed, and refined throughout the analytic process. Table 4 shows the top collocates of “smoker*N*.”

**Table 4.** Top lexical collocates of “smoker* N*” (ranked by mutual information [MI] score) and their themes^a^.

Theme	Top 20 collocates (MI score) sorted in observed themes			
	TIC^b^	HOC^c^	BNC2014^d^	COCA^e^
Smoker* specific categorization	deceased (9.9) adult (9.6) alive (9.5) ASU30^f^ (9.5) former (8.7)	some-day (9.4) former (8.8) never (8.3) nondaily (8.2) cigarette-only (8.2) occasional (8.1) heavier (8.0) african-american (8.0) non (8.0) novice (7.9) current (7.6) ex-smokers (7.5) persistent (7.4)	ex-smokers (16.8) non-smokers (15.8) smoker (13.1) smokers (12.7) non (9.5) heavy (9.3) current (7.5) former (7.1) never (5.6)	nondaily (13.2) two-pack-a-day (12.8) ex-smokers (12.4) nonsmokers (12.1) non-smokers (11.7) late-onset (10.6) nonsmoker (10.2) smokers (9.7) early-onset (9.6) smoker (9.0)
Actors	heir (10.1) dependents (9.3)	sexes (7.9) quitters (7.3)	—^g^	insley (12.2) drinker (8.8) drinkers (8.4)
Products	ultra-low-price (10.0)	non-menthol (7.5)	tobacco (11.0) cigarettes (10.8) cigarette (9.6)	e-cigarettes (9.1) nicotine (8.5) cigarettes (7.5) cigarette (7.4)
Behavior	—	intakes (7.9)	smoking (10.9) smoked (10.9) smoke (9.3)	—
Cessation	assisting (9.1) switch (8.8) convert (8.7)	switched (7.8) tips (7.5)	quit (11.5) help (6.0)	cessation (7.4)
Health	—	HrQol (7.6)	dust (9.5)	methylation (7.9) emphysema (7.6)
Legal	r$1,000 (10.1) alleges (9.3) smoker’s (9.2) antismoking (9.1) estates (9.1) expended (9.0) comprised (8.8)	—	—	—
Measures	—	—	likely (6.6) higher (6.5)	—
Other	convince (9.6) diminished (9.3)	—	—	—

a Asterisks indicate “wildcard” searches, which capture variable word endings, and in “smart” searches, they limit word form searches within a specified part of speech category.

b TIC: Tobacco Industry Corpus.

c HOC: Health Organizations Corpus.

d BNC2014: British National Corpus 2014.

e COCA: Corpus of Contemporary American English.

f ASU30: adult smokers under 30 years.

g Not applicable.

Across all 4 corpora, the plural term “smokers” was used to describe either a class (“smokers that”; raw frequencies: TIC=32, HOC=21, BNC2014=3, and COCA=19) or a group of individuals (“smokers who”; raw frequencies: TIC=995, HOC=731, BNC2014=22, and COCA=176), with the group classification (“who”) used more often.

Otherwise, overlap in the lexical construction of smokers was limited. Of the 23 specific categorizations of “smoker* N” found among the top collocates, only 7 were shared across all corpora: “former,” “never,” “non,” “current,” “ex,” “heavy,” and “regular,” indicating that most high-ranking descriptors were corpus-specific. Different groups, therefore, rely on largely distinct vocabularies when talking about smokers.

#### Tobacco Users

##### Tobacco Industry Corpus

In relation to smokers, the TIC was the only corpus that included references to mortality in the lists of top collocates: “deceased” and “alive” are both top collocates of smoker* N*. Together with the prominence of associated actors in the top collocates, such as “heir” and “dependents,” this shows a tendency toward impersonal descriptions of tobacco users in legal contexts. On the other hand, the classifier “ASU30” (adult smokers under 30 y) positions smokers as consumers. Health-related collocates were absent among the top results.

Several TIC collocates reflect the tobacco industry’s emphasis on regulatory positioning. The term “diminished” appeared in several different contexts, but its key association with “smoker*” was in reference to risk, particularly in the context of regulatory obstacles, for example, “significant risks include our diminished ability to convert adult smokers to our reduced-risk products (RRPs).” Similarly, “convince” was used in reference to a transition to other products, often discussed in the context of regulation and coinciding with calls for “supportive government policy.” Additional TIC-exclusive collocates, such as “assisting” and “convert,” further supported this trend.

We noted the mention of adult smokers’ intentions in these discussions, which suggested that the use of “RRPs” was behaviorally equivalent to smoking, as in “convince all adult smokers who intend to continue smoking to switch to RRPs as soon as possible.”

These constructions simultaneously emphasized smoker autonomy (“convince adult smokers”) while normalizing continued nicotine consumption (“would otherwise continue to smoke”), suggesting a strategic distribution of responsibility between individual choice and the regulatory context.

##### Health Organizations Corpus

The HOC foregrounded clinical and behavioral classifications, including “cigarette-only,” “persistent,” and “nondaily,” and positioned smokers within cessation and research contexts through terms such as “quitters,” “intakes,” and “HrQOL.” Many of these classifications were uncommon in the general English corpora, indicating limited uptake of institutional public health terminology in everyday language use. Despite this clinical orientation, disease-specific collocates were not prominent among the HOC’s top results.

##### General English Corpora: BNC2014 and COCA

In the general English corpora, smokers were frequently referred to through contrastive categorization, particularly in association with “nonsmokers”, and through strong associations with tobacco and nicotine products. Product-related collocates were more prominent here than in either the TIC or HOC, positioning smokers primarily within consumer and behavioral contexts.

References to disease (“emphysema”) and probabilistic risk (“higher”,“likely”) were also present in the general English corpora, which is distinct from both the TIC’s legal-consumer framing and the HOC’s clinical and research-centered categorizations. Table 5 shows the top collocates of “smok*V*.”

**Table 5.** Top 20 lexical collocates of smok* V* (ranked by mutual information [MI] score) and their themes^a^.

	Top 20 collocates (MI score) sorted in observed themes			
	TIC^b^	HOC^c^	BNC2014^d^	COCA^e^
Smok* specific categorization	—^f^	some-day (8.6) day (7.0) don’t (7.0)	smokers (10.7)	nonsmokers (8.8) ex-smokers (8.6) secondhand (8.5) non-smokers (8.3)
Actors	dependents (9.4) men (8.9)	mothers (7.6) anyone (6.9)	—	—
Products	—	snuffing (8.9) sucking (8.9) narghile (7.0)	spliff (12.7) cigars (12.3) fags (12.2) cigarettes (12.2) cigar (11.7) weed (11.7) cigarette (11.5) fag (10.7) pipe (10.5) marijuana (10.3) cannabis (9.7)	menthols (8.4) kools (8.4) luckies (8.3)
Behavior	secondhand (9.5) gateway (8.4)	urge (8.0) smokes (7.5) involuntary (7.1) temporarily (7.0) secondhand (7.0)	smokes (11.2) smoked (10.2) drank (9.5) smoke (9.5) smoking (9.4)	early-experiment (9.7) cigarettes/day (9.7) pack-years (9.6) nondaily (9.6) two-pack-a-day (8.9) pack-a-day (8.9)
Cessation	otherwise (9.2) switch (8.8) continuing (8.7) complete (8.5) completely (8.5)	—	—	cessation (8.3)
Health	respirable (10.6) multidisciplinary (10.3) dangers (10.1) scientifically-substantiated (9.9) ventilated (9.8) injured (9.5)	recode (8.3) machine-smoking (7.5) column (7.3) admitted (7.1) unknown (7.0)	BMI (9.4)	sidestream (10.0) F2RL3 (8.8) varenicline (8.5)
Legal	estates (9.0)	—	—	—
Other	dissuade (10.0) competes (9.4) deserve (8.5)	waking (7.6) fewer (7.0)	deprivation (9.8)	third (8.6)
Measure	25,000 (10.4)	—	—	—
Other nonrelevant	—	—	chimney (9.7)	hoggz (9.9) continus (9.0)

a Asterisks indicate “wildcard” searches, which capture various word endings following a given stem.

b TIC: Tobacco Industry Corpus.

c HOC: Health Organizations Corpus.

d BNC2014: British National Corpus 2014.

e COCA: Corpus of Contemporary American English.

f Not applicable.

### Tobacco Use Behaviors

#### Tobacco Industry Corpus

As with smoker* N*, smoking behaviors (smok* V*) were associated with legal discourses in the TIC. “Dependents” was among the associated actors, reinforcing litigation-related narratives.

Health-related collocates in the TIC, such as “respirable,” “dangers,” and “injured,” were primarily situated within discussions of liability rather than prevention. The collocates “multidisciplinary” and “scientifically-substantiated” reflect a strategic deployment of scientific language in support of regulatory and product positioning.

#### Health Organizations Corpus

In the HOC, smoking behaviors were linked to clinical and population-level concerns. The collocate “mothers” points to maternal and infant health contexts, while collocates such as “recode,” “machine-smoking,” and “unknown” relate to medical research discourse. An exception to this is “admitted,” which relates to the provision of health care related to tobacco use.

#### General English Corpora: BNC2014 and COCA

Similar to the collocates of smoker* N*, smoking behavior (smok* V*) is strongly associated with tobacco and nicotine products. In the BNC2014, smoking behavior is also associated with cannabis products. Brand or product names did not feature in the top collocates in the TIC, but both “Luckies” and “Kools” were top collocates in one of the general English corpora (COCA).

### Use in Context

**Figure 1.**
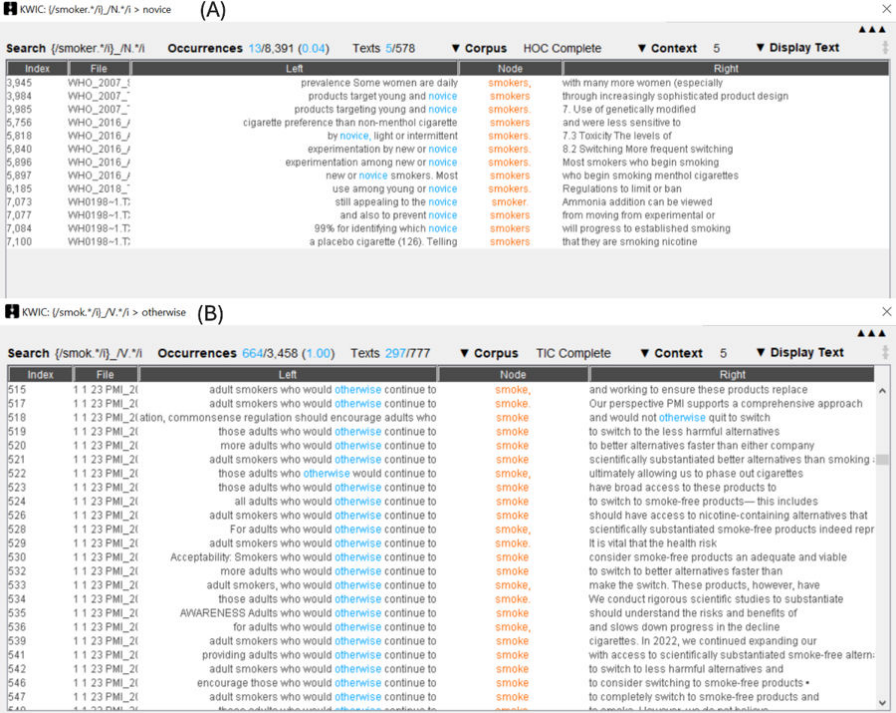
Key Word In Context (KWIC) analysis showing collocations in 2 corpora: (A) “novice” (smoker*N*) in HOC and (B) “otherwise” (smok*V*) in TIC. These results are from a complex “smart search” that includes both a wildcard (open word ending) and tagging for part-of-speech (N* for any noun and V* for any verb). HOC: Health Organizations Corpus; TIC: Tobacco Industry Corpus.

Iterative sorting of collocates into themes permitted the exploration of language patterns that either recurred across corpora or were distinctive to particular organizations. For example, the categorization of some tobacco users as “novice smokers” in the HOC, or the classification of tobacco use behaviors such as “otherwise” in the TIC, as shown in Figure 1.

The HOC and COCA both included the term “novice smoker*,” but this term was exclusive to the WHO in the HOC and found across 3 genres in COCA (fiction, academic, and web). The threshold for this categorization is not clearly described in either case; it is variably used by the WHO, preceded by either “young and” or “new or.”

In the TIC, “otherwise” as a collocate of “smok*V*” was used formulaically in a phrase repeated more or less verbatim from one document to another to talk about a group of people (variably described) “who would otherwise continue to smoke.”

## Discussion

### Principal Findings

Our analysis highlights a high degree of variation in the language used to describe smoking and smokers among the tobacco industry (BAT and PMI), national and international health bodies and services (WHO, NICE, CDC, and NHS), as well as in samples of general English language use (BNC2014 and COCA).

The HOC included more fine-grained classifications for smoker* N* than the other corpora, but each corpus we queried contained descriptors that were not shared with other corpora. This indicates significant and continuing misalignment in the ways in which different groups talk about tobacco use, in spite of calls for change [11,14,15]. It also provides clear evidence of an ongoing “jingle-jangle” problem noted by previous work [11], highlighting key patterns of language held by individual organizations.

The prominence of partially nonoverlapping vocabularies in our analysis, including the differentiation of plural (smokers) and singular (smoker) forms between the corpora, highlights the progress yet to be made in aligning shared understandings of tobacco use risk and determining structural solutions to overcome tobacco-related harms. Indeed, it is evident that addressing tobacco use as a population-level matter is difficult when only 1 group (HOC) frames it as such.

### What Is a Smoker and What Is Smoking?

Frequency analysis highlighted that the plural or singular construction of smoker* as a noun diverged between the general English corpora (BNC2014 and COCA) and the tailor-made corpora: “smokers” were significantly more common in the HOC compared to the TIC, BNC2014, and COCA. The more common reference to smokers in the plural by the HOC, as well as the less frequent use of either “people who” or “people that” (smoke*) across all the corpora, indicates that habits of using person-first phrasing have not yet been established in general English, by health organizations or by the industry [13].

Our results show that BAT and PMI consistently frame smokers as consumers (“adult” and “ASU30”) and as players in legal dramas (“dependents” and “estates”) over dimensions seen in the other corpora (“mothers”). All corpora included categories of behavioral or status descriptions focusing on time or frequency (“never,” “daily,” and “former”). This is an important reminder that tobacco is a commercially driven industry. The characterization of the agency of the business demonstrated a focus on the provision of access to products and information, while the agency of individuals was emphasized in their will to quit or to “continue to smoke.” The characterizations of smoker agency by the TIC were complex, emphasizing the agency of individuals in their phrasing, such as “won’t” and “choose not to,” for example, while deterministically characterizing the smokers’ futures by talking about those who “would otherwise continue to smoke,” presenting continued consumption as both chosen and structurally anticipated.

It was not obvious from our analysis that tobacco use uptake was a central concern for any of the groups. It was reported indirectly by PMI, which noted it as a concern of others regarding its “noncombustible” product portfolio acting as a “gateway to smoking.” Other terms we reported as top collocates, such as “novice,” “early-experiment,” and “early-onset,” may be useful in future work exploring the narrativization of smoking trajectories by different groups and the role of personal agency and discourse in navigating those trajectories.

The high number of product terms in the general English corpora bolsters existing arguments that product variation is a key driver of nebulous understandings of tobacco use behaviors and combustible tobacco products [8]. The prevalence of associated actors and behaviors in the general English corpora also indicates that smokers and smoking are consistently linked to other behaviors, such as drinking, by general audiences. The plural form “smokers” commonly collocates with “drinkers,” which is indicative of a habitual grouping of people according to their behaviors in general English.

Person-first language (people who or that) is not being equally taken up by different groups, calling into question the effectiveness of calls for its use, especially among nonspecialist audiences (those speaking general English).

### Implications for Public Health

The uniquely appalling toll the tobacco industry has had on human health is writ large in our collocational analysis of the TIC; key examples include repeated mention of a legal case focused on a “deceased” smoker and the need to categorize “adult” smokers as a distinct use category (which would not be necessary if there were no nonadult smokers). The data also highlight specific categories of “smoker” that may be a key strategic focus for the industry—ASU30—and confirm key areas of concern for the company, such as PMI’s “diminished ability” to convert adult smokers to their “RRPs,” which was listed as a risk in business reports from 2019 until 2022. This “diminished ability” contrasts with the same organization’s reliance on capacity and resource-based framings in their discussions of tobacco-related harm [38] and highlights how access to policy arenas and an ability to shape regulatory policy are key to addressing business risks for transnational tobacco companies.

In a world where even health-focused organizations like the WHO have been subject to critique for their inconsistent and ambiguous language [39], we must endeavor to remain vigilant to linguistic patterns and the epistemic power of organizational actors. The predictability of downstream impacts of product availability and regulation [7,19] may prove elusive given the lack of common or shared phrasing between consumers, policymakers, and tobacco manufacturers, especially in relation to electronic devices [9]. Our results show that these terminological inconsistencies persist across groups, even when the analysis is highly tailored to focus on a single topic (tobacco). These differences are potentially dangerous at both the individual and population levels. They have been shown to lead to misunderstandings of product harm [4,40] and undermine the consumers’ ability to make informed choices about tobacco product use. A powerful tobacco industry can leverage information gaps and misalignments of understanding to its own advantage [41-44] and begin to embed expectations for those who use or regulate tobacco—including expectations about product choice and product use trajectories, for example, in the portrayal of the use of “RRPs” as behaviorally equivalent to smoking.

Our work shows that linguistic clarity and a common understanding of what forms of tobacco use constitute “smoking,” as well as what it means to use tobacco (to be “a smoker”), are lacking between key groups. Given that information-seeking is one of many processes of interpersonal communication that can impact personal health [45], and the recognized association between exposure to tobacco-related media content and tobacco use [46], understanding how flows of information in the public domain interact with (or indeed contradict) one another is paramount to the reduction of structural health inequalities. This includes the reduction of informational access barriers and working collaboratively to overcome imbalances resulting from the distortion of “sensemaking resources” [47], whether by intent or by design.

Public information campaigns are one mechanism by which international-level health organizations attempt to mitigate the deleterious effects of tobacco use, and yet we have noted that these organizations use highly variable language in their communications. The tobacco industry, too, fosters its own characterizations of what smoking is and who smokers are. In the background, we have general English—a multitude of different genres (books, news articles, and so on) where individuals are exposed to different characterizations of tobacco products and their use. By highlighting the linguistic variation that characterizes public-domain discussions of tobacco use across key groups, we hope that our work can contribute to public health efforts to stem flows of misinformation and help disarm a tobacco industry with a history of concealing known harms associated with tobacco products and manipulating policy design and implementation for their own ends.

### Limitations

As our summaries of the corpora show, there was a significant variance in the size of the corpora we deployed in our analyses, and while we endeavored to mitigate the effect of this by reporting relative frequencies in addition to raw frequencies and by foregrounding other relatively adjusted scores such as mutual information, we recognize that this variance impacts the data. We worked to match our corpora to one another as best we could, including in size and date range, but it is important to point out that the dates of our tailored corpora and the prebuilt corpora do not exactly overlap.

We intentionally excluded personal narratives focused on individuals from the TIC corpora to protect the privacy of named individuals, but existing publications show that these can be valuable in exploring personal narratives relating to tobacco use, especially with regard to reducing hermeneutical injustices through the inclusion of different sense-making practices and resources [47].

The inclusion of documents from organizations within a limited time frame is inherently constrained with regard to the carrying forward of research conclusions. All corporate entities are subject to change, including significant internal restructuring, which can involve reductions in the available specialized expertise used to shape public policy. Such changes may pose a threat to expertise-led information provision in key informational spaces available to consumers. Monitoring linguistic change specific to certain disease areas or product landscapes, such as tobacco, could therefore be helpful in shaping the design of population-relevant and impactful public information campaigns that can be safeguarded against such organizational volatility.

### Future Work

Possible valuable future work could seek to query product-specific narratives or focus on the development of narratives in web-based communication to determine how personal technology use, such as information-seeking or dispersed content production, has impacted the information landscape. It would also be valuable to compare discourse across document types within TIC—for example, between customer-facing webpages and more formal corporate or legal reports—to assess how genre and audience influence narrative strategies. Tracing the development of specific terms over time and considering their distribution across genres could also provide insight into the dispersion of industry-constructed language into popular usage and identify key informational asymmetries and key waypoints in their evolution.

## Abbreviations

ASU30: adult smokers under 30 years
BAT: British American Tobacco
BNC2014: British National Corpus 2014
CDC: Centers for Disease Control and Prevention
COCA: Corpus of Contemporary American English
HOC: Health Organizations Corpus
NHS: National Health Service
NICE: National Institute for Health and Care Excellence
PMI: Philip Morris International
RRP: reduced-risk product
TIC: Tobacco Industry Corpus
WHO: World Health Organization
